# Editorial: Hospital management and healthcare policy: financing, resourcing and accessibility

**DOI:** 10.3389/fpubh.2024.1440141

**Published:** 2024-07-23

**Authors:** Bing-Long Wang, Jay Shen, Hanadi Y. Hamadi, Jeffrey P. Harrison, Thomas T. H. Wan

**Affiliations:** ^1^School of Health Policy and Management, Chinese Academy of Medical Sciences & Peking Union Medical College, Beijing, China; ^2^Center for Health Disparities and Research, School of Public Health, University of Nevada in Las Vegas, Las Vegas, NV, United States; ^3^Department of Health Administration, Brooks College of Health, University of North Florida, Jacksonville, FL, United States; ^4^School of Global Health Management and Informatics, University of Central Florida, Orlando, FL, United States

**Keywords:** integrating innovation, equity, comprehensive research, hospital management, advancing public health

As the editors of “Frontiers in Public Health,” Research Topic “*Hospital Management and Healthcare Policy: Financing, Resourcing and Accessibility*,” we are pleased to introduce this Research Topic, which highlights pivotal research across multiple critical categories: Healthcare policy, Hospital Management, Healthcare Financing, Healthcare Information, and Health Insurance. Each of these domains represents a cornerstone in the architecture of effective public health systems worldwide. This collection of studies provides invaluable insights into the challenges and innovations shaping the future of global health.

## Healthcare policy

Healthcare policy remains at the forefront of addressing disparities and optimizing care. One compelling study, “*Association of geographical disparities and segregation in regional treatment facilities for black patients with aneurysmal subarachnoid hemorrhage in the United States*,” (Kabangu et al.) underscores the urgent need to address regional inequalities in healthcare access. Additionally, “*The socioeconomic burden of spinal muscular atrophy in Saudi Arabia: A cross-sectional pilot study*” (Alotaibi et al.) illuminates the profound economic impact of rare diseases on families and health systems.

Cost-effectiveness analyses, such as those conducted for tobacco cessation interventions in Thailand and atezolizumab treatment for non-small-cell lung cancer in the UK, offer critical evaluations of resource allocation in health interventions. The study on corruption types during the COVID-19 pandemic across Western and Central-Eastern health systems provides a stark reminder of the systemic challenges that can undermine public health efforts.

Moreover, the research on inpatient respiratory conditions before and during the COVID-19 pandemic reveals significant changes in healthcare demands and patient profiles. A systematic review and meta-analysis on financial toxicity among cancer patients highlights the economic burdens faced by individuals across different income countries, emphasizing the need for equitable healthcare financing.

## Hospital management

Hospital management plays a vital role in ensuring the efficiency and effectiveness of healthcare delivery. The comparative study of human resource management in the top five global hospitals offers valuable lessons in achieving operational excellence. In China, a DEMATEL-Based network analysis model identifies key factors affecting the mental health and performance of hospital administrators working from home, reflecting the new challenges posed by remote work environments.

A machine learning-based cross-sectional study on the healthcare costs of cardiovascular disease in China and a micro-costing analysis of suspected lower respiratory tract infection care in a French emergency department provide deep dives into the financial intricacies of healthcare services. Qualitative insights from Chinese hospital leaders on Joint Commission International (JCI) accreditation and a game theory-based approach to optimizing internal control in public hospital supply chains further contribute to the discourse on hospital management.

## Healthcare financing

In healthcare financing, innovative approaches to risk governance, such as those addressing fraudulent reimbursement of patient consultation fees in China, are critical in maintaining the integrity of healthcare systems. The consideration of patient perspectives in economic evaluations of health interventions in Canada represents a progressive step toward patient-centered care. Additionally, the cross-sectional comparison of healthcare delivery and reimbursement between segregated and non-segregated communities in Hungary sheds light on the implications for addressing social and economic disparities of healthcare access.

## Healthcare information

The effective use of healthcare information systems is essential for modern healthcare. Studies on incentive mechanisms for sharing and using electronic health records (EHR) in medical consortiums and the application of telemedicine systems for older adult postoperative patients in community settings highlight the transformative potential of digital health innovations. These studies from China emphasize the importance of performance evaluation and feasibility in implementing new health technologies.

## Health insurance

Health insurance is a fundamental pillar of universal health coverage. The appraisal of universal health insurance and maternal health services utilization before and after the implementation of Jaminan Kesehatan Nasional (JKN) in Indonesia provides critical insights into the impact of health insurance policies on healthcare access and outcomes. This study exemplifies the ongoing efforts to achieve health equity through comprehensive insurance schemes.

In conclusion, this Research Topic brings together a diverse array of studies that collectively advance our understanding of healthcare policy, hospital management, healthcare financing, healthcare information, and health insurance. Each article offers its unique contributions to the ongoing discourse on how to improve public health systems globally. As we continue to face new challenges and opportunities in public health, the research presented here serves as a vital resource for policymakers, practitioners, and scholars dedicated to fostering healthier communities worldwide.

## Author contributions

B-LW: Conceptualization, Validation, Writing – original draft, Writing – review & editing. JS: Conceptualization, Writing – review & editing. HH: Conceptualization, Writing – review & editing. JH: Conceptualization, Writing – review & editing. TW: Conceptualization, Supervision, Validation, Writing – review & editing.

